# Imaging studies for predicting hematoma expansion: from traditional imaging signs to artificial intelligence-based multimodal fusion

**DOI:** 10.3389/fneur.2026.1843413

**Published:** 2026-06-26

**Authors:** Jie Wu, Jinping Sheng, Yu Xiao, Fa Wu, Pingping He, Rui Jiang, Zhiwei Zuo, Peng Wang

**Affiliations:** 1 Department of Radiology, The General Hospital of Western Theater Command, Chengdu, Sichuan, China; 2 Department of Respiratory and Infectious Diseases, The General Hospital of Western Theater Command, Chengdu, Sichuan, China

**Keywords:** artificial intelligence, deep learning, hematoma expansion, imaging markers, intracerebral hemorrhage, multimodal fusion, radiomics

## Abstract

Hematoma expansion (HE) is a critical and modifiable event following acute intracerebral hemorrhage (ICH). Predicting HE accurately can inform individualized treatment and improve patient outcomes. This review systematically outlines the evolution of imaging-based HE prediction. We first define the core concepts of traditional HE, revised HE (rHE), and ultra-early hematoma growth (uHG). We then summarize predictive studies that employ traditional imaging markers, such as the computed tomography angiography (CTA) spot sign, non-contrast CT (NCCT) signs, and combined clinical-imaging scoring systems. Subsequent sections focus on AI-driven methodologies, encompassing radiomics, deep learning, and multi-task learning. The discussion extends to precision prediction through multimodal data fusion and subgroup analyses based on hemorrhage location and onset time. Finally, we address persistent challenges, including model interpretability, generalizability, and translational gaps, and suggest future directions involving federated learning, explainable AI, dynamic prediction, and closed-loop decision systems. This review offers a structured framework to guide both clinical practice and future research.

## 1 Introduction

Intracerebral hemorrhage (ICH) is a devastating stroke subtype, with a 30-day mortality of 30–40% and frequent severe neurological deficits (1, 2). In China, with an aging population and a high prevalence of risk factors such as hypertension, the burden of stroke is disproportionately heavy, with a substantially higher proportion of intracerebral hemorrhage compared to Western populations (3, 4). Recent global burden analyses have consistently highlighted that China bears the largest absolute burden of ICH worldwide, accounting for nearly half of all global incident cases, and this trend is projected to persist through the coming decades (5). Hematoma expansion (HE) is a key influential and modifiable factor. Approximately 20–30% of patients experience HE within 24 h of onset, which is strongly associated with early neurological deterioration, disability, and increased mortality (6, 7). Therefore, preventing HE—via blood pressure control, coagulation correction, or hemostatic agents—is a major therapeutic target. Accurate prediction of HE is essential for targeted intervention. HE is influenced by onset time, hematoma volume and morphology, anticoagulant use, blood pressure, and inflammatory and coagulation status (8, 9). Imaging markers have evolved from the CTA spot sign (10) and NCCT signs (11–13), to multiphase CTA (14) and ultraearly hematoma growth (uHG) (15), underscoring imaging’s central role.

Recently, radiomics and deep learning have enabled high-throughput extraction of subvisual image features, outperforming traditional markers (16, 17). Meanwhile, the concept of HE has evolved: revised hematoma expansion (rHE) includes intraventricular hemorrhage growth to better reflect total bleeding burden (18). The field now focuses on multimodal fusion of clinical, laboratory, and imaging data for individualized prediction (19–21).

Despite progress, challenges remain in model generalizability, interpretability, standardization, and clinical translation (22). This review summarizes imaging advances for HE prediction, traces the shift from traditional markers to AI-driven methods, analyzes multimodal fusion and subgroup analyses, and discusses challenges and future directions for clinical practice.

## 2 Core concepts and definitions

Before delving into prediction methods, it is essential to clarify the definition and evolution of the core outcome measure—“hematoma expansion.” A clear and unified conceptual framework is a prerequisite for understanding and comparing different studies.

### 2.1 Traditional definition of hematoma expansion (HE) and its limitations

HE is defined as a significant increase in hematoma volume on follow-up imaging. The most widely used criterion is absolute growth >6 mL or relative growth >33% (6, 9). However, this definition has limitations. It ignores intraventricular hemorrhage (IVH) growth and is a static, binary measure that fails to capture bleeding dynamics. For instance, it treats a small (10 to 13.5 mL) and a large (50 to 60 mL) expansion identically, despite differing clinical impacts. This has prompted revised concepts.

### 2.2 Revised hematoma expansion (rHE): a new perspective incorporating intraventricular hemorrhage growth

IVH, a common complication, can also expand after initial imaging (delayed IVH). Yogendrakumar et al. proposed “revised HE (rHE),” combining traditional HE criteria with IVH growth (e.g., “≥6 mL or ≥33% or any new IVH”) (18). In the PREDICT-ICH study, rHE improved sensitivity for predicting 90-day poor outcome versus traditional HE (63.8% vs. 45.7%) with minimal specificity loss, better reflecting total bleeding burden, marking a reconceptualization of HE. Since then, an increasing number of studies have adopted rHE as the primary outcome measure (23–25), further validating its value in clinical prognostic assessment.

### 2.3 Ultraearly hematoma growth (uHG): from static volume to dynamic rate

In addition to focusing on the “total amount” of bleeding, researchers have also begun to emphasize the “rate” of bleeding. Knowing whether a hematoma will eventually expand is far less important than understanding the rate at which it expands, especially for guiding ultra-early interventions. To this end, Rodriguez-Luna et al. introduced the concept of “ultraearly hematoma growth (uHG),” defined as the baseline hematoma volume divided by the time from symptom onset to the first imaging study (unit: mL/h) (15).

**Figure 1:**
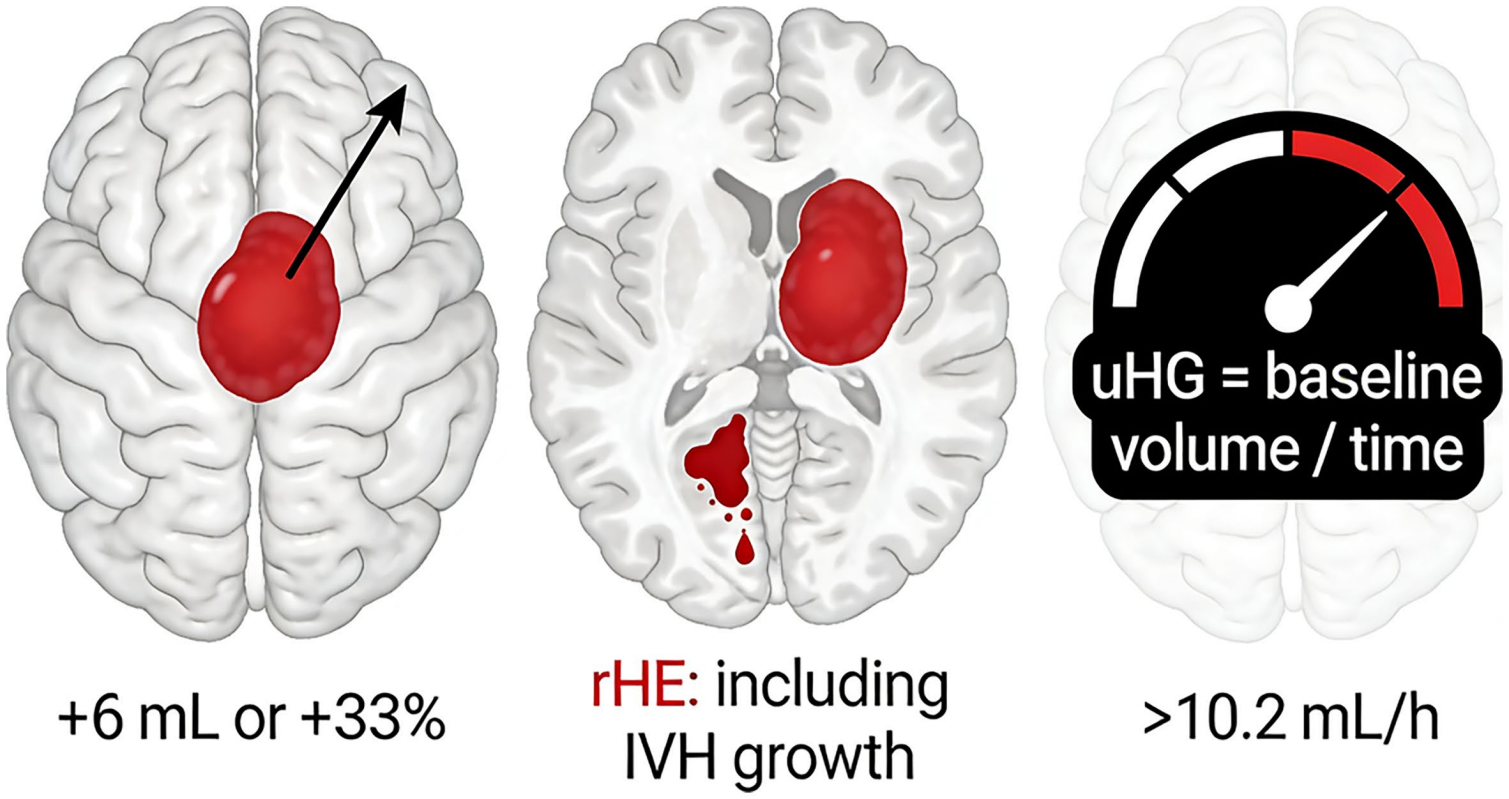
Evolution of core concepts. The evolution of hematoma expansion (HE) definitions. From left to right: Traditional HE defined by absolute (>6 mL) or relative (>33%) volume increase; Revised HE (rHE) incorporating intraventricular hemorrhage (IVH) growth; Ultraearly Hematoma Growth (uHG), calculated as baseline volume divided by time from onset, with a critical threshold of >10.2 mL/h indicating a high-risk bleeder. Original figure created for this review.

Their study found that uHG was significantly faster in patients who eventually developed HE, early neurological deterioration, death, and poor long-term outcomes. More importantly, uHG > 10.2 mL/h was one of the strongest predictors of these adverse outcomes, with predictive performance even superior to baseline hematoma volume alone. Subsequent studies not only confirmed the value of uHG in predicting traditional HE and prognosis (26) but also extended its application to predicting revised hematoma expansion (27) and found that the subgroup of “fast bleeders” identified by uHG might be the greatest potential beneficiaries of intensive blood pressure reduction therapy (15, 28). The introduction of uHG marks a deepening of our understanding of HE from a static judgment of “whether it occurs” to a dynamic assessment of “how fast it occurs,” providing a powerful tool for ultra-early risk stratification and individualized treatment. (as illustrated in Figure 1).

## 3 Prediction studies based on traditional imaging markers

Before the widespread application of artificial intelligence, researchers primarily relied on the visual observation of radiologists to identify specific signs on images associated with HE risk. These traditional imaging markers constitute the first cornerstone of HE prediction research and continue to play an important role in clinical practice today.

### 3.1 CTA spot sign: from “gold standard” to refined assessment

The CT angiography spot sign is defined as one or more, punctate or serpiginous, foci of enhancement within a hematoma on CTA source images, with density significantly higher than that of the surrounding hematoma and discontinuous with adjacent normal vessels. Since Wada et al. first systematically described its predictive value (10), the spot sign has been confirmed by numerous studies as one of the most powerful and reliable imaging markers for predicting HE. It represents direct imaging evidence of active, ongoing arterial or arteriolar bleeding, i.e., contrast extravasation into the hematoma.

As research has deepened, the assessment of the spot sign has evolved from simple “presence/absence” to “refined characterization.” Rodriguez-Luna et al. used multiphase CTA to categorize the appearance of the spot sign into arterial, venous, and delayed phases (14). They found that the earlier the phase in which the spot sign appeared (i.e., more arterial), the higher the frequency and extent of significant HE. This finding reveals the dynamic nature of the spot sign, suggesting that its timing may reflect the activity level and bleeding rate of the bleeding source, enabling more refined risk stratification for HE. Although the spot sign has excellent predictive efficacy, its application depends on CTA examination. Moreover, its high negative predictive value, especially after excluding anticoagulation-related bleeding, can effectively screen out low-risk patients who do not require frequent follow-up imaging (29).

### 3.2 The family of NCCT markers: black hole sign, blend sign, island sign, hypodensities, etc

As CTA is not routine, NCCT-based markers are widely studied given their availability. These markers attempt to capture information related to active bleeding and instability from the density and morphological characteristics of the hematoma.

**Figure 2:**
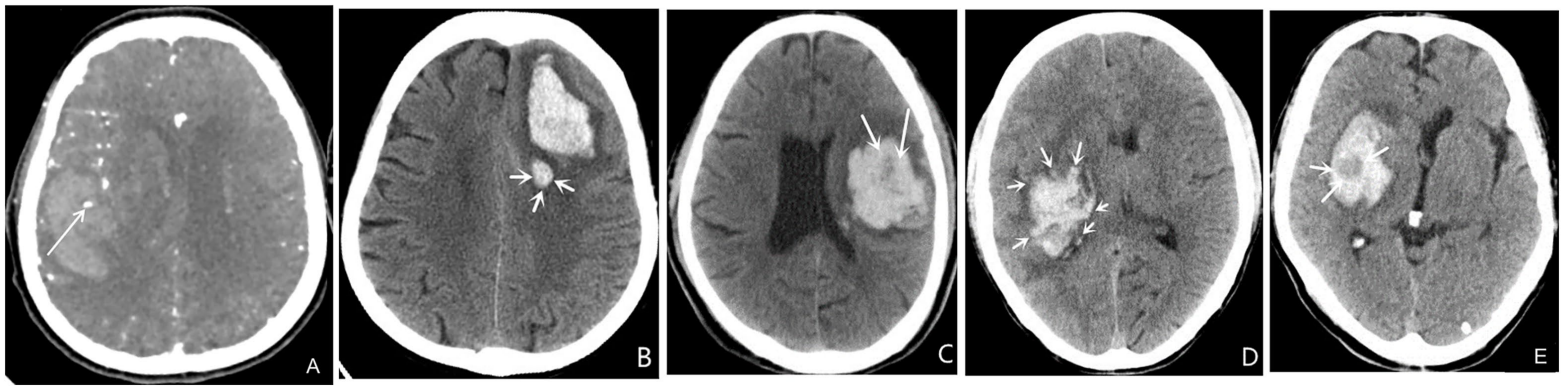
The family of traditional imaging markers. A summary of traditional imaging markers for predicting hematoma expansion. The central image highlights the CTA spot sign **(A)**. NCCT markers including the satellite sign **(B)**, swirl sign **(C)**, island sign **(D)**, and black hole sign **(E)** each visualized with arrow highlight for easy identification. This composite image was created by the authors and includes representative clinical cases and illustrations derived or adapted from our previously published work (21). Used with permission. Original figure created for this review.

As early as 2009, Barras et al. (11) proposed a semi-quantitative scale for assessing hematoma density heterogeneity and shape irregularity, finding that density heterogeneity (rather than shape irregularity) was an independent predictor of HE, laying the foundation for subsequent research. Since then, a series of vividly named NCCT markers have been successively proposed and validated, as shown in Figure 2:

- Blend sign: Refers to the presence of two adjacent regions within the hematoma with markedly different densities, a clear boundary, and a density difference >18 Hounsfield units (30).
- Black hole sign: Refers to a relatively hypodense “black hole” enclosed within a hyperdense hematoma, with a clear boundary between the “black hole” and the surrounding hematoma (31).
- Island sign: Refers to the presence of at least three independent, scattered small hematoma “islands” separate from the main hematoma, or the main hematoma is partially connected to these small islands (32).
- Swirl sign: Refers to the presence of round, linear, or irregularly shaped hypodense or isodense areas within the hematoma, suggesting active bleeding or unclotted blood (33).
- Hypodensities: A broader concept referring to any hypodense region within the hematoma margins (34).

A landmark meta-analysis by Morotti et al. confirmed all these markers significantly predicted HE and poor outcome, with the island sign strongest (OR = 7.87) (13). The same group also led efforts to standardize their detection and reporting (35, 36). Subsequent studies further found that the diagnostic performance of these markers may be influenced by the onset-to-CT time; for instance, hypodensities have the highest sensitivity in the ultra-early phase (<2 h), while irregular shape performs better in late presenters (>6 h) (37).

### 3.3 Scoring systems integrating clinical and imaging markers

The predictive power of a single marker is limited, so researchers naturally turned to integrating multiple independent predictors into a single scoring system to achieve more convenient and comprehensive risk stratification. Based on large prospective or retrospective cohorts, several classic HE prediction scoring systems have been developed.

- 9-point Prediction Score: Developed by Brouwers et al., this score is based on four predictors: warfarin use, CTA spot sign, onset-to-CT time (≤6 h vs. > 6 h), and baseline ICH volume (9). Higher scores were associated with higher rates of HE, and the score performed well in an independent validation cohort (C-statistic 0.77), providing a practical tool for individualized treatment and clinical trial design.
- PREDICT Score: Proposed by Huynh et al. based on data from the PREDICT study, this scoring system is more complex, incorporating multiple variables such as spot sign number, onset time, warfarin use/INR, GCS, and NIHSS, and derived the PREDICT A/B scores, showing improved discrimination compared to the 9-point score (37).
- BAT Score: This score is primarily based on NCCT markers, including Blend sign, Any hypodensity, and Time (onset-to-CT time), and is valued for its simplicity and independence from CTA{Citation}.

The emergence of these scoring systems marked the transition of HE prediction from single markers to multi-factor integration. However, these scores are mostly based on traditional logistic regression models, and their performance is limited to a few pre-selected variables. An external validation study conducted in an Asian cohort (38) found that while these scores had acceptable discrimination, their calibration and overall performance still had room for improvement, notably pointing out that scores incorporating the CTA spot sign (PREDICT and 9-point) outperformed the score without it (BRAIN) (38), reaffirming the core value of the spot sign.

## 4 The new paradigm of artificial intelligence-driven imaging prediction

Artificial intelligence technologies, represented by deep learning, have revolutionized HE prediction. Unlike traditional methods that rely on human visual identification and predefined features, AI can automatically learn higher-dimensional and more complex patterns from massive amounts of imaging data, with predictive performance significantly surpassing traditional paradigms.

### 4.1 Radiomics: mining deep quantitative features from images

Radiomics extracts high-dimensional quantitative features (e.g., shape, texture) from medical images. Xie et al. (16) were among the first to apply radiomics to HE prediction. They extracted numerous radiomics features from NCCT images of 251 ICH patients and constructed a radiomics score model. The results showed that this model was significantly superior to traditional radiological models (e.g., blend sign, black hole sign) in predicting HE, and they found that hematomas that expanded tended to have larger baseline volume, more irregular shape, more heterogeneous composition, and coarser texture—features quantified by radiomics that perfectly corroborated the pathophysiological mechanisms. Chen et al. further confirmed the superiority of radiomics models in a larger cohort (1,153 patients) and constructed a clinical-radiomics nomogram that achieved optimal predictive performance (19).

The value of radiomics has also been confirmed by meta-analysis. Jiang et al. conducted a meta-analysis of 10 studies (1,525 patients) showing that the pooled AUC for NCCT-based radiomics models predicting HE was 0.80 (39). Network meta-analysis further indicated that the predictive ability of radiomics models was superior to most NCCT biomarkers. Furthermore, radiomics has been applied to predict revised hematoma expansion (rHE) and to compare 2D and 3D features. Chen et al. found that 2D radiomics features performed comparably to 3D features in predicting HE, and given their higher computational efficiency, they might be more suitable for emergency settings (40).

### 4.2 Deep learning: achieving end-to-end automated prediction

**Figure 3:**
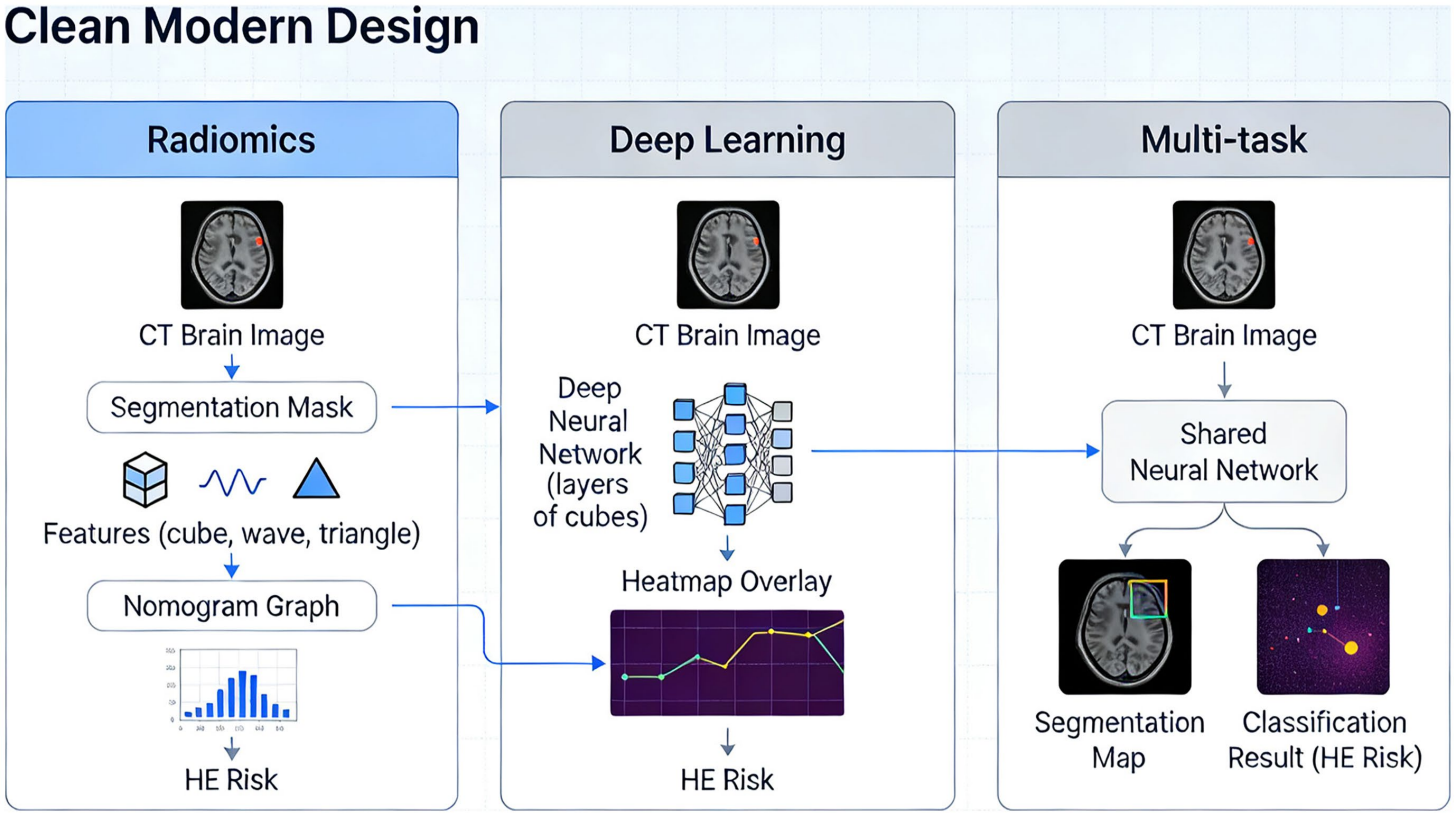
The new paradigm of AI-driven prediction. A comparative flowchart of three AI approaches for hematoma expansion prediction. Panel 1 (Radiomics) uses handcrafted feature extraction and a nomogram. Panel 2 (Deep Learning) employs an end-to-end neural network to generate a predictive heatmap. Panel 3 (Multi-task Learning) jointly optimizes hematoma segmentation and HE risk prediction for improved performance. Original figure created for this review.

Deep learning, particularly convolutional neural networks (CNNs), has taken automation to new heights. It requires no manual segmentation or feature extraction; it can take raw CT images directly as input and output the probability of HE, achieving true “end-to-end” prediction, as depicted in Figure 3.

Zhong et al. (17) developed a deep learning model and compared its performance with traditional NCCT markers and the BAT score. In a validation set of 266 patients, the deep learning model achieved the highest C-statistic (0.80), significantly outperforming individual NCCT signs (e.g., black hole sign, blend sign) as well as logistic models based on hematoma volume and the BAT score. Teng et al. trained an AI model using NCCT images from over 1800 patients, also demonstrating that its specificity and sensitivity for predicting HE were far superior to manually labeled NCCT signs, with a negative predictive value as high as 95.9%, meaning it could very reliably rule out low-risk patients (41). Further advances include attention-based U-Net/ResNet hybrids for full automation (AUC 0.92) (42), single-slice CNNs for efficiency (43), and integrated clinical-deep feature models (AUC 0.92) (44).

### 4.3 Multi-task learning: synergistic optimization of segmentation and prediction

Multi-task learning is an advanced strategy in deep learning where a single model learns multiple related tasks simultaneously. In HE prediction, the most typical multi-task learning model performs hematoma segmentation and HE classification concurrently.

The underlying logic is that the hematoma segmentation task forces the model to learn “low-level” spatial information such as the precise boundaries, shape, and location of the hematoma, which is crucial for the “high-level” HE classification task. By sharing network parameters, the two tasks can mutually reinforce and co-optimize, resulting in final feature representations that contain both accurate localization information and discriminative features relevant to HE risk.

The study by Lee et al. (20) is an exemplary application of multi-task learning. Their “multi-task learning model” achieved excellent diagnostic performance in the hematoma detection task (AUC 0.99). Building on this, the model integrated image features with clinical data to construct an “integrated model” that significantly outperformed the image-only model (AUC 0.76) and the clinical-only model (AUC 0.81) in HE prediction (AUC 0.83). The study by Ning et al. followed a similar approach, demonstrating that their 2D deep learning model (based on ResNet-101) performed better in predicting revised hematoma expansion (rHE) than traditional clinical-radiological models and combined models based on handcrafted radiomics features (45). These studies collectively show that integrating segmentation and prediction within a single framework through multi-task learning not only improves prediction efficiency but also enhances ultimate predictive performance through information complementarity between tasks.

### 4.4 Head-to-head performance comparison: from visual signs to artificial intelligence

To move beyond a mere catalogue of techniques and provide a truly critical synthesis, a direct comparison of predictive performance is essential. The trajectory from traditional markers to AI reveals not just a methodological shift, but a quantum leap in predictive accuracy, which is best illustrated through quantitative measures. Table 1 provides a structured, head-to-head comparative summary of the discriminative power of various models, primarily measured by the Area Under the Curve (AUC).

**Table 1:** Comparative performance of representative models for hematoma expansion prediction.

Model type	Specific model/sign	Predictors	AUC (approx.)	Key characteristics and limitations	Refs.
Traditional scoring	9-Point score	Warfarin, spot sign, time, volume	~0.77	Simple, well-validated; dependent on CTA spot sign.	(9)
	PREDICT A/B score	Spot sign, Time, Warfarin/INR, GCS, NIHSS	>9-Point	Higher complexity; better discrimination but relies heavily on CTA.	(38)
	BAT score	Blend sign, Any Hypodensity, Time	~0.70–0.75	Simplified, fully NCCT-based; best immediate utility in emergency settings.	(17)
Individual NCCT sign	Black Hole Sign	Hypodense region within hematoma	-	Low sensitivity, high specificity; simple visual assessment.	(32)
	Blend Sign	Hematoma with liquid/solid parts	-	Moderate sensitivity; quantitative definitions improve reliability.	(12)
	Island Sign	≥3 scattered small hematomas	-	High specificity (OR = 7.87); reflects complex active bleeding.	(33)
Radiomics	Clinical-Radiomics Nomogram	Rad-score, Age, Location	~0.82	Quantifies texture heterogeneity; requires segmentation. Outperforms clinical models alone.	(19)
	Pooled Radiomics Model	NCCT-based features	0.80 (pooled)	Pooled from meta-analysis; significant improvement over single NCCT signs.	(40)
Deep learning (DL)	DL Model (Zhong et al.)	Raw NCCT	~0.80	End-to-end; superior to BAT score and single NCCT signs.	(17)
	DL Model (Teng et al.)	Raw NCCT	-	High NPV (95.9%); reliable rule-out test.	(42)
	Attention ResNet-34 (Ma et al.)	Raw NCCT	~0.93	High performance on specific (hypertensive) ICH cohort.	(43)
Multimodal fusion	Joint 2.5D DL Model (Peng W et al.)	2.5D DL, Radiomics, clinical, signs	~0.93	The highest benchmark to date; integrates all data dimensions.	(21)

AUC, Area Under the Receiver Operating Characteristic Curve; OR, Odds Ratio, NPV, Negative Predictive Value.

As evidenced in Table 1, a clear performance gradient exists. Early scoring systems like the BAT score, while entirely non-enhanced CT-based, achieved AUCs in the 0.70–0.75 range, a performance ceiling strongly tied to the limits of human visual perception and linear statistical models (17). The transition to AI-driven radiomics shattered this ceiling by extracting thousands of mathematically-defined texture and shape features imperceptible to the naked eye. The pooled AUC of 0.80 from a meta-analysis of radiomics models (40) marks a definitive and statistically significant improvement over any single NCCT marker, as confirmed by Morotti et al.’s landmark meta-analysis on traditional markers (13).

Deep learning models have further consolidated this advantage, demonstrating AUCs ranging from 0.80 to over 0.93 (17, 21, 43). The fully automated, end-to-end pipeline eliminates inter-rater variability and manual segmentation errors. A study by Teng et al. highlighted the clinical utility of this paradigm, reporting a negative predictive value of 95.9%, making it a powerful tool to confidently exclude low-risk patients (42). The current state-of-the-art is represented by multimodal fusion models, which integrate deep imaging features with clinical, laboratory, and classical radiological data to achieve AUCs above 0.93, approaching the performance ceiling for a static, single-timepoint prediction task (21, 46).

However, this comparison is not meant to advocate for a wholesale abandonment of traditional markers. The critical advantage of many NCCT signs and scoring systems lies in their simplicity, real-time availability, and inherent interpretability. A clinician can understand why the BAT score is high by simply looking at the scan. This “glass-box” nature fosters immediate clinical trust and decision-making at the bedside. In contrast, a deep learning model’s “why” requires post-hoc explainability techniques, which are still maturing. The future, therefore, is not competition but integration, as articulated in the multimodal fusion strategy, where the strengths of each paradigm are leveraged to build systems that are simultaneously more accurate, generalizable, and interpretable.

## 5 Towards precision: multimodal information fusion and subgroup analysis

As understanding of the pathophysiological mechanisms of HE deepens, coupled with the development of AI technologies, researchers increasingly recognize that single imaging markers or clinical indicators cannot fully capture the complexity of HE. HE is the result of the interaction between the “lesion” (imaging characteristics of the bleed) and the “host” (the patient’s systemic condition, underlying diseases, coagulation function, inflammatory status). Therefore, a comprehensive prediction paradigm that fuses multi-source information has emerged and quickly become a frontier hotspot in the field. This chapter systematically elaborates on the latest advances in multimodal fusion strategies and explores how subgroup analyses based on bleeding location and other features can further propel HE prediction towards individualized precision medicine.

### 5.1 Integration of multimodal information: from clinical-imaging combination to AI fusion models

**Figure 4:**
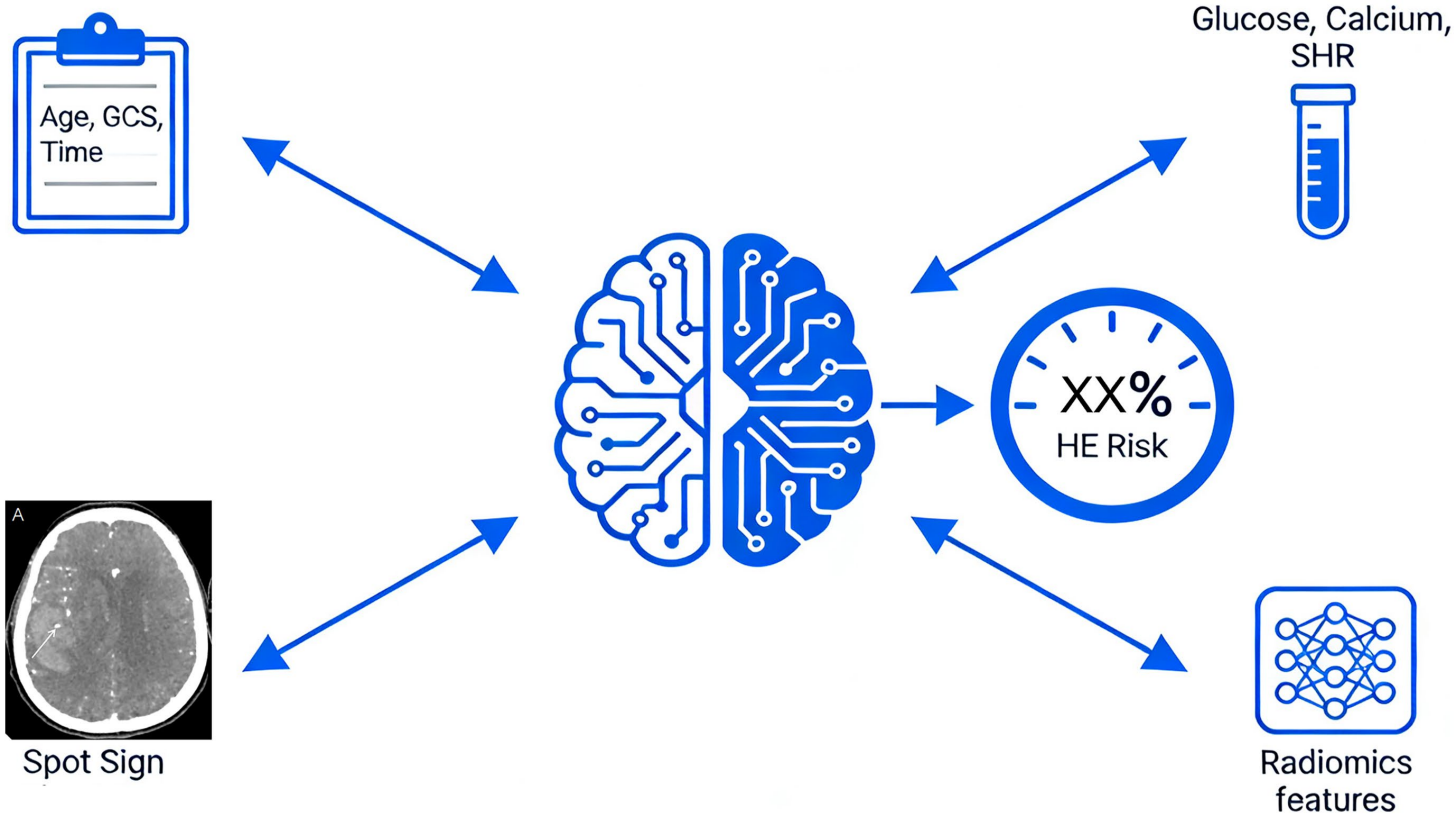
Towards precision: multimodal information fusion. A multimodal fusion AI model integrating diverse data sources for precise HE risk prediction. The central model combines clinical parameters, laboratory results, traditional imaging signs, and AI-extracted radiomics features. The output is a personalized HE risk score (e.g., xx%), with key predictive features highlighted via SHAP analysis for interpretability. Original figure created for this review.

The core idea of multimodal fusion is to integrate information from different dimensions—including clinical parameters (e.g., age, GCS score, onset time), laboratory indicators (e.g., blood glucose, calcium, inflammatory markers), traditional imaging signs, and advanced imaging features (radiomics, deep learning features)—in order to achieve predictive efficacy surpassing any single modality, as shown in Figure 4.

Early studies used logistic regression to combine clinical factors with imaging markers (e.g., radiomics scores or traditional signs), consistently showing improved performance over single-modality models (19, 47). Machine learning models like SVMs further captured non-linear relationships (48). Notably, SHAP analysis identified key predictive features, enhancing interpretability (49). Deep learning fusion strategies, including multi-task learning (20, 42) and integrated clinical-imaging networks45, have achieved AUCs around 0.83–0.92 (44). The most comprehensive models now fuse 2.5D deep features, clinical data, radiological signs, and radiomics, achieving AUCs up to 0.929 (21, 50). Furthermore, incorporating pathophysiological biomarkers—such as stress hyperglycemia ratio (SHR) (51), serum calcium/glucose (52), or inflammatory scores (53)—has further enhanced predictive accuracy and biological plausibility.

In summary, multimodal information fusion has become a consensus strategy for improving HE prediction. From simple logistic regression to complex deep learning ensemble models, from clinical and imaging data to the continuous addition of biochemical and inflammatory markers, research in this field is progressing rapidly along a path that is more comprehensive, more intelligent, and more interpretable.

### 5.2 Subgroup analysis: from population-level prediction to individualized precision assessment

Building on multimodal fusion, researchers have begun to realize that HE prediction models should not be “one-size-fits-all.” The mechanisms, risk factors, and prognostic impact of HE may differ significantly across etiologies and locations of ICH. Therefore, conducting in-depth analyses on specific subgroups is a key step towards individualized precision prediction.

Bleeding location is one of the most extensively studied subgroup variables. Leasure et al. (46), in a secondary analysis of the ATACH-2 trial data, found that while intensive blood pressure reduction was associated with reduced HE risk overall, this association was significant in patients with basal ganglia hemorrhage but not observed in those with thalamic hemorrhage. This finding suggests that the specific deep nuclei involved (basal ganglia vs. thalamus) may modulate the effect of blood pressure intervention on HE. Li et al. (26) further refined this perspective, finding that involvement of the posterior limb of the internal capsule (PLIC) was a strong predictor of HE in patients with deep ICH (OR = 2.73), indicating that even within the broad “deep” category, involvement of different anatomical structures has markedly different effects on HE risk.

Studies comparing deep and lobar ICH have also revealed important differences. Zhao et al. (54) found that although deep ICH seemed more prone to HE, lobar ICH had larger baseline hematoma volumes. More critically, the HE threshold associated with poor outcome was lower in deep hemorrhages (30% vs. 50%), suggesting that deep brain tissue is less tolerant of hematoma expansion, and even small-volume expansion can lead to severe consequences. Kuohn et al. (55) in a post-hoc analysis of the FAST trial, also found that despite lobar hemorrhages exhibiting higher rates of HE and early neurological deterioration, deep ICH was associated with worse outcome after adjusting for confounders like hematoma volume, likely due to its proximity to eloquent brain structures. Furthermore, Morotti et al. (56) discovered that subarachnoid extension (SAHE) was independently associated with increased HE risk only in lobar ICH (OR 6.00), but not in non-lobar ICH. This reveals a location-specific predictor of HE.

Besides location, onset time is another important dimension for subgroup analysis. Morotti et al. focused on patients with “unclear symptom onset (USO),” a common clinical scenario but often excluded from clinical trials (57). They found that the frequency of HE in USO patients was not significantly different from those with clear onset, and HE was independently associated with poor outcome. Importantly, NCCT hypodensities remained an independent predictor of HE in USO patients, providing a key basis for early risk stratification in this special population. Additionally, subgroup analysis based on bleeding rate has shown tremendous clinical value. Qi et al., reanalyzing ATACH-2 data, found that in the subgroup of “fast bleeders” (uHG > 5 mL/h), intensive blood pressure reduction was associated with a significantly lower risk of HE (20.6% vs. 31.0%), whereas no such effect was seen in slower bleeders (28). This suggests that the “fast-progressing” subgroup, identified by ultraearly hematoma growth rate (uHG), may be the greatest potential beneficiaries of intensive blood pressure reduction therapy, perfectly exemplifying the translational concept from “prediction” to “guiding intervention.”

In summary, subgroup analysis is propelling HE prediction research from the “macro population” towards the “micro individual.” Whether it is bleeding location, onset time, or bleeding rate, these factors profoundly modulate the risk of HE and the response to treatment. Future predictive models must be capable of capturing this heterogeneity, providing personalized risk assessments and intervention recommendations for patients in different subgroups.

## 6 Clinical translation and future perspectives

Although research on predicting HE has made remarkable progress over the past two decades, translating these achievements into clinical practice still faces a series of serious challenges. Confronting these challenges and exploring potential breakthrough paths are the core tasks for the future development of this field.

### 6.1 Current challenges: model interpretability, generalizability, and external validation

Three core challenges hinder clinical translation: limited model interpretability, poor generalizability across centers, and lack of unified validation benchmarks. These issues reduce clinical trust and comparability between models.

### 6.2 From prediction to intervention: individualized treatment strategies based on risk stratification

The goal of prediction is to guide intervention. Future models should use causal inference to estimate treatment effects and identify patients who benefit most from hemostatic or blood pressure therapies.

### 6.3 Future directions: federated learning for multi-center data, multimodal foundation models, dynamic prediction

Based on the above analysis, we propose the following three most promising future research directions:

- Direction 1: Multi-Center Generalizable Models Based on Federated Learning. To address the issues of data silos and insufficient generalizability, federated learning offers a highly promising technological pathway. It allows multiple medical institutions to collaboratively train a global model without sharing raw patient data (preserving data privacy). Each center’s data is used only for local model updates, with only encrypted model parameters uploaded to a central server for aggregation. In this way, the final global model can learn from diverse data distributions across different regions, devices, and populations, potentially significantly improving model generalizability and robustness. Building a large-scale HE prediction model based on multi-center federated learning would be a major step towards clinical application in this field.
- Direction 2: Interpretable Models Integrating Multimodal Data and Pathophysiological Mechanisms. To bridge the gap between “black box” and “trust,” future research needs to incorporate interpretability as a core element of model design, rather than a post-hoc add-on. Specific pathways include:

1. Application of Concept Activation Vectors (TCAV): Answer not only “which pixels are important” but also “whether the model makes decisions based on high-level clinical concepts like ‘hematoma irregularity’ or ‘presence of spot sign’.”
2. Design of Inherently Interpretable Models: Explore using attention mechanisms or graph neural networks (GNNs) so that the model’s reasoning process is naturally interpretable. For example, GNNs can explicitly model spatial relationships between different regions within the hematoma, and their decision-making path is itself a quantitative analysis of hematoma morphology.
3. Deep Integration of Pathophysiological Parameters: Incorporate biomarkers reflecting key pathological processes (e.g., blood–brain barrier leakage rate Kᵗʳᵃⁿˢ measured by DCE-MRI) as inputs or constraints for the model, aligning the model’s learning process with biological mechanisms, thereby grounding its predictions more firmly in science (58, 59).

- Direction 3: Dynamic Prediction and Early Warning Systems Based on Longitudinal Data

Current “static” prediction models cannot capture the dynamic evolution of a patient’s condition after admission. The future direction is to build dynamic prediction models based on longitudinal data. Continuous blood pressure monitoring, hourly laboratory tests, and scheduled or unscheduled follow-up CT scans constitute valuable time-series data. Using recurrent neural networks (RNNs), long short-term memory networks (LSTMs), or Transformers, we can learn the dynamic patterns of these data and update the predicted probability of HE in real-time. Imagine an early warning system embedded in the hospital information system that continuously “monitors” the condition of every ICH patient and alerts clinicians when the predicted risk of HE rises sharply, creating a valuable “golden window” for intervention.

### 6.4 Towards clinical decision support systems: opportunities and obstacles

Ultimately, all these technologies should be integrated into a user-friendly, workflow-integrated clinical decision support system (CDSS). This system should automatically fetch data from electronic health records and picture archiving systems, run predictive models in the background, and present results and recommendations to physicians in an intuitive manner (e.g., risk dashboards, visualization heatmaps).

However, the path from research to a functional CDSS is fraught with obstacles:

- Technical and Engineering Integration: Seamlessly embedding complex AI models into existing hospital information systems requires strong engineering capabilities and interdisciplinary teams.
- Regulation and Ethics: As software influencing medical decisions, a CDSS requires stringent regulatory approval. Issues of model fairness, transparency, and accountability also need in-depth ethical consideration.
- Clinical Acceptance: Even if the technology matures, overcoming the psychological barrier and gaining trust from physicians accustomed to traditional experience will require time and evidence from rigorous studies.
- Health Economics Evaluation: What is the cost-effectiveness of introducing an AI-CDSS? Can it improve patient outcomes while reducing overall healthcare costs? These are the primary concerns of payers and healthcare administrators.

**Figure 5:**
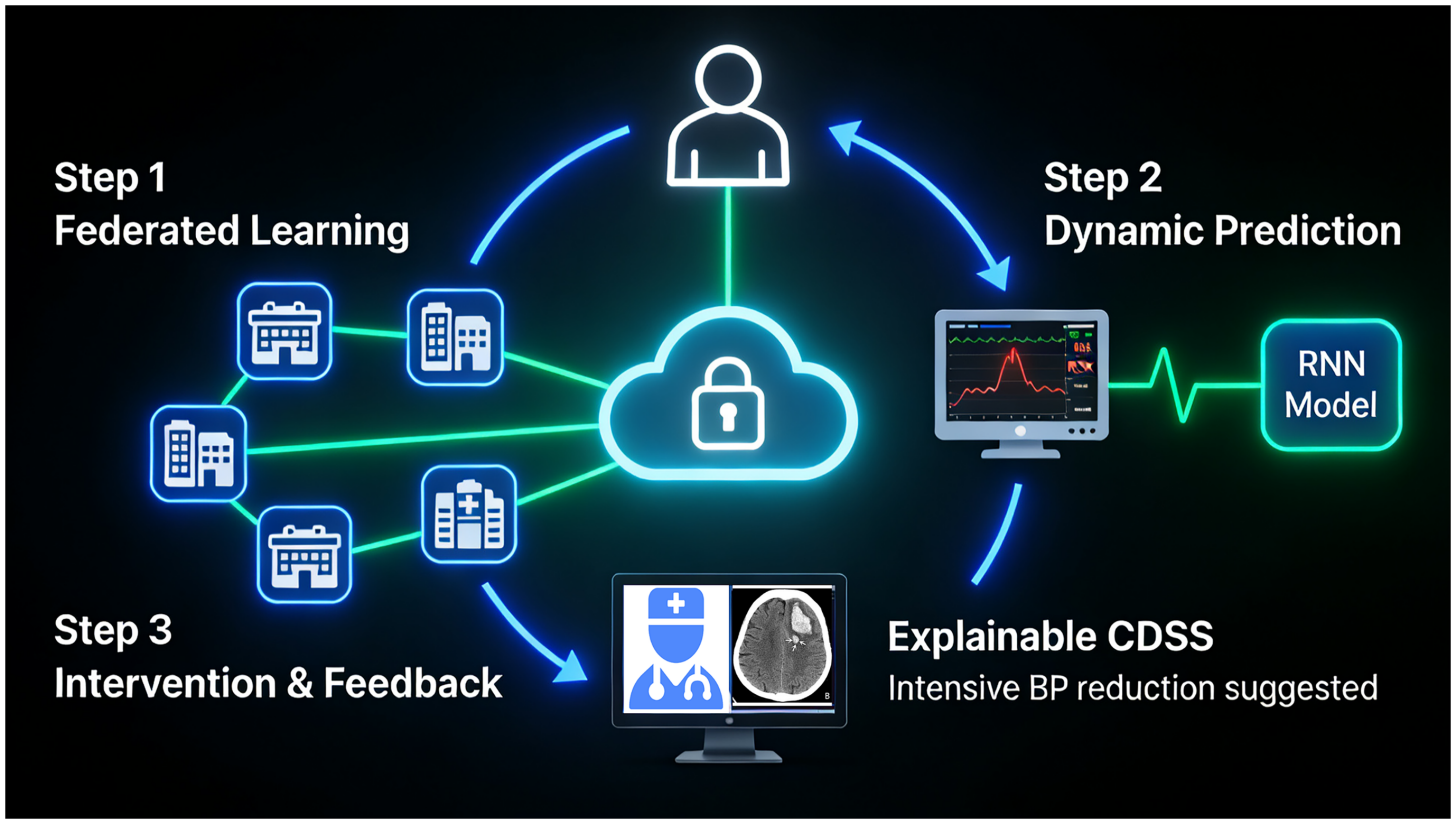
Future perspectives: a closed-loop system from prediction to intervention. A futuristic closed-loop clinical decision system for stroke management. This system leverages federated learning for privacy-preserving model training, dynamic prediction using recurrent neural networks (RNN), an explainable clinical decision support system (CDSS) to guide interventions, and continuous feedback from patient outcomes to refine the model, forming a complete learning cycle. Original figure created for this review.

Although the road ahead is long, the direction is clear as illustrated in Figure 5. Through multidisciplinary collaboration and tackling these challenges one by one, we have reason to believe that AI-based models for predicting HE will eventually emerge from the laboratory, integrate into clinical practice, and contribute meaningfully to improving the outcomes of patients with intracerebral hemorrhage.

## 7 Conclusion

Hematoma expansion is a critical and treatable event in acute ICH, and its accurate prediction supports personalized treatment. Imaging prediction has evolved from traditional CTA and NCCT markers to integrated scoring systems. Revised definitions and uHG improve risk stratification. AI methods including radiomics, deep learning, and multi-task learning deliver stronger predictive performance. Multimodal fusion and subgroup analysis enable precision prediction. Key challenges include model interpretability, generalizability, and clinical translation. Future directions include federated learning, explainable AI, dynamic prediction, and closed-loop decision systems. The field is shifting toward clinical value-driven care to improve outcomes for ICH patients.
